# Effects of Different Types of Dietary Fibers on Lipid Metabolism and Bile Acids in Weaned Piglets

**DOI:** 10.3390/ani13203266

**Published:** 2023-10-19

**Authors:** Yaolian Hu, Dongting He, Bing Yu, Daiwen Chen

**Affiliations:** Key Laboratory of Animal Disease-Resistant Nutrition, Ministry of Education, Animal Nutrition Institute, Sichuan Agricultural University, Ya’an 625014, China; 2020114015@stu.sicau.edu.cn (Y.H.);

**Keywords:** dietary fiber, fructooligosaccharides, bile acids, gut microbiota, weaned piglets

## Abstract

**Simple Summary:**

Dietary fiber, as an essential nutrient, is very important for the health of animals and it is necessary to study the effects of dietary fiber on animal physiology and its mechanism. In the swine industry, the excessive deposition of body lipids in pigs has a negative impact on the lean meat rate. Previous studies showed that dietary fibers can inhibit lipid accumulation in pigs during the growing-finishing period, but it remains unclear what the effect of fiber is on the lipid metabolism in weaning pigs. Thus, this study investigated how dietary fiber regulates lipid metabolism in piglets. We found that dietary fibers had abilities to reduce lipid synthesis in piglets. Additionally, increasing evidence suggests that bile acids are associated with body lipid metabolism, and some bile acids (hyocholic acid species) are the key factors for the regulation of lipid metabolism. In this study, dietary fibers, especially fructooligosaccharides, elevate the levels of hyocholic acid species possibly via the gut microbiota and its enzymes. The bile acids altered by dietary fiber supplementation are closely correlated with lipid metabolism. These results deepen our understanding of dietary fibers and bile acids, and provide a reference for the application of them in the livestock industry.

**Abstract:**

The aim of this study was to investigate the effects of dietary fiber on the serum biochemistry, bile acid profile, and gut microbiota in piglets. Twenty-four pigs (initial body weight: 10.53 ± 1.23 kg) were randomly divided into three treatments with eight replicate pens of one pig per pen for 21 d. The dietary treatments consisted of the following: (1) a fiber-free diet (NS); (2) a fiber-free diet + 3% fructooligosaccharides (SI); (3) a fiber-free diet + 3% dietary fiber mixture (fructooligosaccharides, long-chain inulin, and microcrystalline cellulose at the ratio 1:1:1; MIX). The results showed that compared with the NS group, the 3% SI diet reduced the serum total cholesterol (TC) concentration of the piglets (*p* < 0.05). The metabolomics results showed that the 3% SI diet increased the level of taurohyocholic acid (THCA) and α-muricholic acid, and the 3% MIX diet increased the level of THCA and cholic acid (*p* < 0.05). The use of 3% SI or MIX decreased the glycodeoxycholic acid (GDCA) level in the bile of the piglets (*p* < 0.05). The correlation analysis shows that the GDCA was positively related to the TC. The 16S rRNA gene sequencing results showed that UCG-002 and Holdemanella were enriched in the SI group, while Bacteroides was enriched in the MIX group. The microbial function prediction indicated that SI supplementation tended to elevate the relative abundance of gut bacteria capable of expressing bile acid-metabolizing enzymes. To sum up, the regulatory effect of dietary fiber on lipid metabolism is related to bile acids in piglets. Compared with MIX, SI is more likely to regulate bile acids through the gut microbiota.

## 1. Introduction

Dietary fibers, which are commonly found in plant-based food and feed materials, are important for the growth, metabolism, and immunity of animals [1,2]. Previous studies noted that dietary fibers can be classified into insoluble and soluble fibers according to their solubility [3]. Insoluble fibers include microcrystalline cellulose and lignin, while soluble fibers refer to fructooligosaccharides and long-chain inulin [4,5]. In the swine industry, the excessive deposition of body lipid in pigs has a negative impact on the lean meat rate and carcass quality [6]. Wu et al. presented that the inclusion of 5% microcrystalline cellulose decreased the total and LDL cholesterol in the plasma of growing pigs [4]. Zhou et al. noted that inulin addition decreased the gestational body weight gain and fat accumulation in sows [7]. Grela et al. also found that inulin addition decreased the backfat thickness in finishing pigs [8]. These results indicate that dietary fibers can inhibit lipid accumulation and improve the carcass quality in growing-finishing pigs. Some studies noted that the content of body lipids within weaning pigs would easily affect body lipid deposition in the growing-finishing period [9,10]. However, it is not fully clear how dietary fiber affects the lipid metabolism in weaning pigs.

Previous studies noted that the mechanisms of different dietary fibers to regulate lipid metabolism were different, in that insoluble fiber can increase satiety, while soluble fiber affects host health via the gut microbiota and its metabolites [8,11,12]. Secondary bile acids are the important metabolites derived from the intestinal microbiota, and they have been shown to affect gut health and host metabolism [13,14,15]. Bile acids are divided into primary bile acids, which are produced by hepatic cholesterol, and secondary bile acids, which are converted from primary bile acids by gut microbiota [16,17]. Wang found that there was a significant difference in the bile acid profiles between low birth weight (LBW) and normal birth weight neonatal piglets, and oral administration of ursodeoxycholic acid (UDCA), a secondary bile acid that was reduced in the LBW piglets, can inhibit intestinal inflammation and promote the intestinal health of LBW piglets [18]. Another study pointed out that dietary supplementation with a bile acid mixture (chenodeoxycholic acid, hyodeoxycholic acid, and hyocholic acid) can improve the colonic function of intrauterine growth retardation (IUGR) piglets [19]. This suggests that bile acids are closely associated with the gut health of piglets. In addition, Kuang et al. presented that the group of gut microbiota-modified bile acids, hyodeoxycholic acid (HDCA) species, had abilities to alleviate non-alcoholic fatty liver disease (NAFLD) through activating farnesoid X receptor (FXR) [20]. The researchers observed the expressions of the genes related to lipid synthesis (*SREBP-1c*, *ACC*, *FAS*) were downregulated after intervention with HDCA in the high-fat diet-fed mice. Previous research has indicated that hepatic FXR activation can suppress the expression of *ChREBP* and *SREBP-1c* via small heterodimer partner (SHP) [20]. These results imply the capacity of certain secondary bile acids to regulate hepatic lipid synthesis through FXR. Recent evidence indicates a connection between dietary fiber and bile acids, with dietary fiber demonstrating the ability to regulate bile acid levels [21]. A new experiment explored whether fructooligosaccharides supplementation in mice fed a Western-style diet improved the related indicators of glucose metabolism via the gut microbiota and secondary bile acids [21]. It remains unclear whether the effect of dietary fiber on lipid metabolism is related to bile acids. Thus, in this study, we used fructooligosaccharides and a dietary fiber mixture (fructooligosaccharides, long-chain inulin, and microcrystalline cellulose at the ratio 1:1:1) to investigate their effect on lipid metabolism, bile acid profiles (composition and content), and lipid metabolism in weaning pigs.

## 2. Materials and Methods

The protocol for animal care was approved by the Animal Ethical and Welfare Committee of Sichuan Agricultural University (20210223).

### 2.1. Materials

Fructooligosaccharides and microcrystalline cellulose were purchased from Changzhou Yangsen Biotechnology Co., Ltd. (Guangzhou, China). Long-chain inulin, which is derived from chicory, was obtain from Fengning Ping’an High-tech Industrial Co., Ltd. (Chengde, China).

### 2.2. Diets and Experimental Design

The experiment was conducted using a single-factor experimental design, and twenty-four Duroc × Landrace × Yorkshire pig (initial body weight: 10.53 ± 1.23 kg; ages: 33 ± 1 days old) were randomly divided into 3 treatments with 8 replicate pens of 1 pig per pen. The dietary treatments consisted of the following: (1) a fiber-free diet (NS); (2) a fiber-free diet + 3% fructooligosaccharides (SI); (3) a fiber-free diet + 3% dietary fiber mixture (fructooligosaccharides, long-chain inulin, and microcrystalline cellulose at the ratio 1:1:1; MIX). The trial lasted for 21 days. The chemical compositions of basal diets are listed in Table 1. All pigs were housed in an environmentally controlled animal facility and allowed access to water and feed ad libitum. The study calculated the average daily gain (ADG), average daily feed intake (ADFI), and the feed to gain ratio (F/G). Feeds were provided three times a day at 08:00, 14:00, and 20:00.

### 2.3. Sample Collection

At the end of the experiment, after 12 h fasting, blood samples were obtained and serum was separated by centrifugation at 3500× *g* for 10 min at 4 °C, and these pigs were anaesthetized by intramuscular injection of Zoletil (0.1 mL/kg body weight) and slaughtered. The serum, bile, liver tissue, and colonic contents were collected and stored at −80 °C.

### 2.4. Biochemical Analysis

Serum total cholesterol (TC) (catalogue number: A111-1-1), triglyceride (TG) (catalogue number: A110-1-1), high-density lipoprotein cholesterol (HDL-C) (catalogue number: A112-1-1), low-density lipoprotein cholesterol (LDL-C) (catalogue number: A113-1-1), and total bile acids (TBA) (catalogue number: E003-2-1) were determined using kits provided by Nanjing Jiancheng Bioengineering Institute (Nanjing, China).

### 2.5. Analysis of Bile Acids in Bile by Ultra-Performance Liquid Chromatography Tandem Mass Spectrometry (UPLC-MS/MS)

The analysis of the bile acids profile was completed by Shanghai Zhongke New Life Biotechnology Co., Ltd., Shanghai, China. Fifteen substances, including cholic acid-d4 (TRC, C432603), chenodeoxycholic acid-d4 (TRC, C291902), deoxycholic acid-d5 (TRC, D232647), glycocholic acid-d5 (TRC, G641352), α-hyodeoxycholic acid-d5 (TRC, H998102), and others, were used as isotope internal standards for quantification. The unlabeled bile acid standards, such as cholic acid (C1129), hyodeoxycholic acid (H3878), α-muricholic acid (C1890-000), taurohyodeoxycholic acid (C0890-000), ω-muricholic acid (C1889-000), and others, were obtained from Sigma-Aldrich Corporation (St Louis, MO, USA) or Steraloids Inc. (Newport, RI, USA). The bile samples were thawed at 4 °C and 100 μL aliquots were mixed with 10 μL of isotope internal standard and 500 μL of cold methanol to remove the protein. The mixture was centrifuged for 20 min (14,000× *g*, 4 °C), and the supernatant was dried in a vacuum centrifuge. The dried sample needed to be re-dissolved in 100 μL methanol/water (1:1, *v*/*v*) and adequately vortexed, and then centrifuged (14,000× *g*, 4 °C, 15 min) [22]. The reacquired supernatants were collected for LC-MS/MS analysis. Analysis was performed using an UHPLC (Waters Ltd., Milford, MA, USA) coupled online to 5500 QTRAP Mass Spectrometry (AB SCIEX, Foster City, CA, USA).

LC–MS quantification of bile acid metabolites was performed using six-point standard curves. Isotope internal standard diluted in a relevant matrix matched to the analytical sample was used for absolute quantification. Data acquisition and processing were carried out using Multiquant software 3.0.3 (AB SCIEX).

### 2.6. Real-Time Quantitative PCR

Total RNA of liver homogenates was extracted using RNAiso Reagent (TaKaRa) and the RNA samples were reversely transcribed into cDNA using PrimeScript RT reagent kit with gDNA Eraser (TaKaRa, Dalian, China) according to manufacturer’s instructions. The concentration and purity of total RNA isolation were determined before reverse transcription PCR by DU 640 UV spectrophotometer detection (Beckman, Brea, CA, USA). RT-qPCR was performed using the 7900 HT Fast Real-time PCR system (384-cell standard block) and the SYBR Green PCR Master Mix. The primers used were listed in Table 2. β-actin was used as an internal control to normalize the expression of the selected genes and the relative expression levels of these target genes were calculated by using the 2^−∆∆Ct^ method [23].

### 2.7. 16S rRNA Gene Sequencing

The DNA of colonic content was extracted by E.Z.N.A.^®^ Stool DNA Kit. For 16S rRNA sequencing, the DNA samples were sent to the Novogene Company (Beijing, China). Gut microbiota was analyzed based on the 16S rRNA gene V3–V4 sequencing of DNA samples. The V3–V4 region of the 16S rRNA gene was amplified using universal primers, including the forward primer 341F (5′-CCTAYGGGRBGCASCAG-3′) and reverse primer 806R (5′-GGACTACNNGGGTATCTAAT-3′). PCR reactions were performed using 15 μL of Phusion^®^ High-Fidelity PCR Master Mix (New England Biolabs, Ipswich, MA, USA), 2 μM of forward and reverse primers, and approximately 10 ng of template DNA. The thermal cycling protocol consisted of an initial denaturation step at 98 °C for 1 min, followed by 30 cycles of denaturation at 98 °C for 10 s, annealing at 50 °C for 30 s, and elongation at 72 °C for 30 s. A final extension step was performed at 72 °C for 5 min. The quantification and qualification of PCR products were performed by electrophoresis on 2% agarose gels. Sequencing libraries were generated using the TruSeq^®^ DNA PCR-Free Sample Preparation Kit (Illumina, San Diego, CA, USA), and the library was sequenced on an Illumina NovaSeq platform.

The Quantitative Insights into Microbial Ecology2 (QIIME2) software (Version QIIME2-202202) was used to carry out the Bioinformatics analysis of sequencing data. Microbial function analysis was conducted using the phylogenetic investigation of communities by reconstruction of unobserved states (PICRUST2) based on 16S rRNA gene amplicon sequences. The functional annotation of PICRUST2 prediction was obtained based on several gene family databases such as Kyoto Encyclopedia of Genes and Genomes11 (KEGG) orthologs (KOs) and Enzyme Commission numbers [24,25].

### 2.8. Statistical Analysis

Data was analyzed by one-way ANOVA procedures using IBM SPSS Statistics 23 software, Chicago, IL, USA. The replicate (each replicate in one cage) was taken as an experimental unit for all statistical analyses. Tukey’s multiple comparison test was conducted to determine the statistical differences. For bile acids analysis, the metabolites with a relative standard deviation (RSD) of quality control (QC) samples less than 30% were selected for subsequent statistical analysis. A Spearman’s correlation analysis was used to clarify the relationship of bile acids profiles and phenotypic parameters. Results show the mean of all pigs per group and standard error of means (SEM) of all pigs, in which *p* < 0.05 was considered statistically significant.

## 3. Results

### 3.1. Growth Performance

From Table 3, the piglets fed 3% SI diets showed a lower ADG (*p* < 0.05) and a higher F/G (*p* < 0.05) compared to NS.

### 3.2. Serum Biochemistry

Compared with NS, dietary supplementation with 3% SI tended to reduce the serum TC concentration of the piglets (*p* = 0.08) and the 3% dietary fiber mixture significantly reduced the serum total bile acid levels of the piglets (*p* < 0.05) (Table 4).

### 3.3. Hepatic Lipid Metabolism-Related Genes Expression

**Figure 1:**
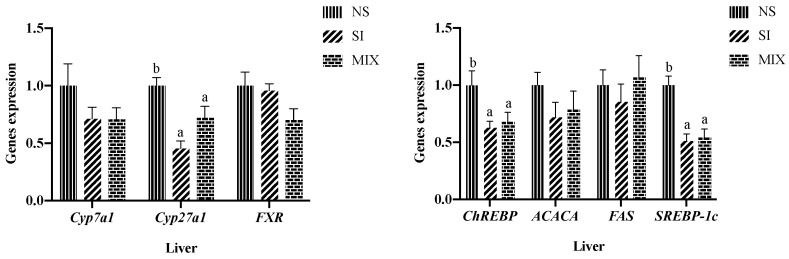
Effect of different treatments on lipid metabolism-related genes expression levels in liver. Eight piglets per treatment. Mean values followed by different letters within a row are significantly different (*p* < 0.05). *SREBP-1c*, sterol regulatory element-binding protein-1c; *FAS*, fatty acid synthase; *ACACA*, acetyl-CoA carboxylase; *ChREBP*, carbohydrate response element binding protein; *FXR*, farnesoid X receptor; *Cyp7a1*, cholesterol 7 alpha-hydroxylase; *Cyp27a1*, sterol 27-hydroxylase.

Feeding the piglets with 3% SI or 3% dietary fiber mixture decreased the mRNA expression level of carbohydrate response element binding protein (*ChREBP*), sterol regulatory element-binding protein-1c (*SREBP-1c*), and sterol 27-hydroxylase (*Cyp27a1*) (*p* < 0.05) (Figure 1).

### 3.4. Bile Acid Profiles of Bile

**Figure 2:**
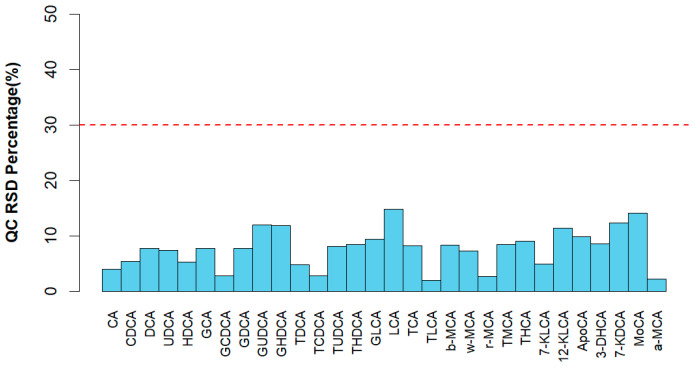
The RSD distribution of bile acids in QC samples.

From Figure 2 and Table 5, the 3% SI diet increased the level of THCA and α-muricholic acid (α-MCA), and the 3% MIX diet increased the level of THCA and cholic acid (*p* < 0.05). The 3% SI or MIX diets decreased the level of GDCA in the bile of the piglets (*p* < 0.05).

### 3.5. Spearman’s Correlation Analysis

**Figure 3:**
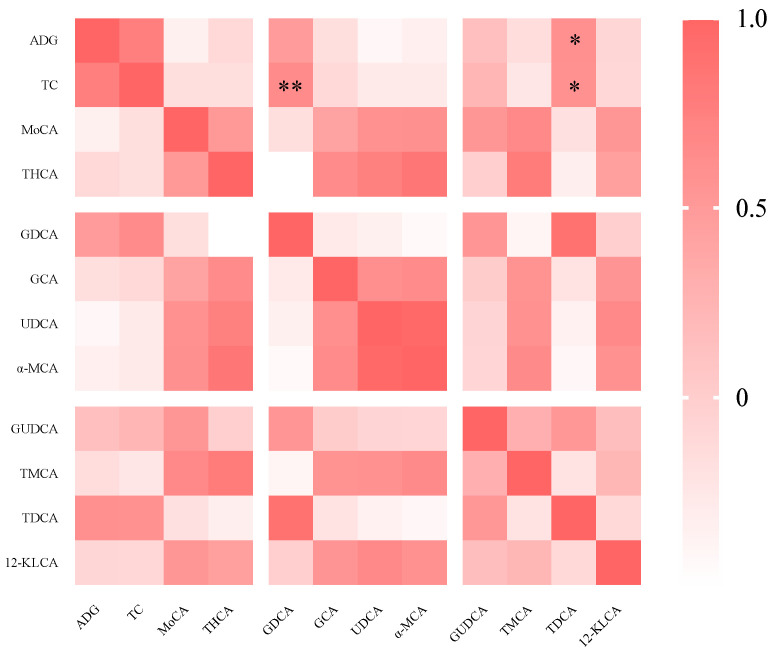
The correlation between bile acid profiles in bile and phenotypic parameters. * *p* < 0.05; ** *p* < 0.01.

The correlation analysis indicated that there was a significant positive correlation between the serum TC concentration and the levels of GDCA (*p* < 0.01) and TDCA (*p* < 0.05) in the bile of the piglets (Figure 3).

### 3.6. The Composition and Function of Gut Microbiota

**Figure 4:**
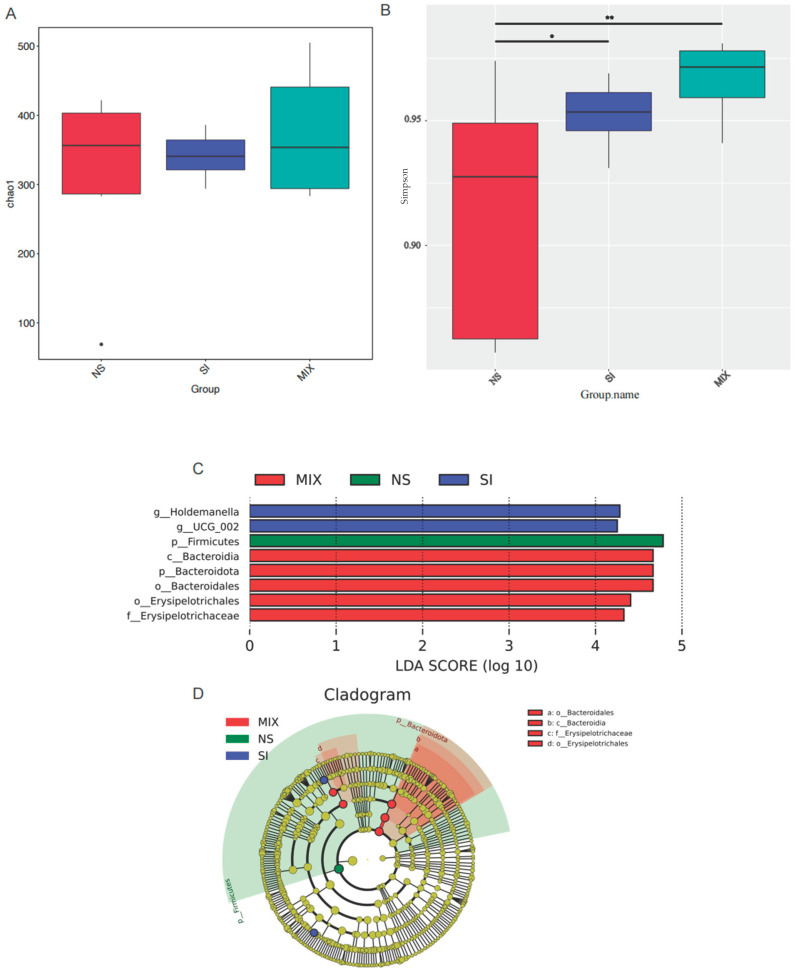
Effect of different treatment on gut microbiota in colon of piglets. (**A**) Chao1 index (**B**) Simpson’s index showing the effect of dietary fiber on species abundance and/or diversity of colon in piglets. (**C**) Linear discriminant analysis (LDA) effect size (LEfSe) analysis to identification of bacteria in the SI and MIX group. (**D**) Taxonomic cladogram obtained from LEfSe analysis of 16S sequence. *, *p* < 0.05; **, *p* < 0.01.

**Figure 5:**
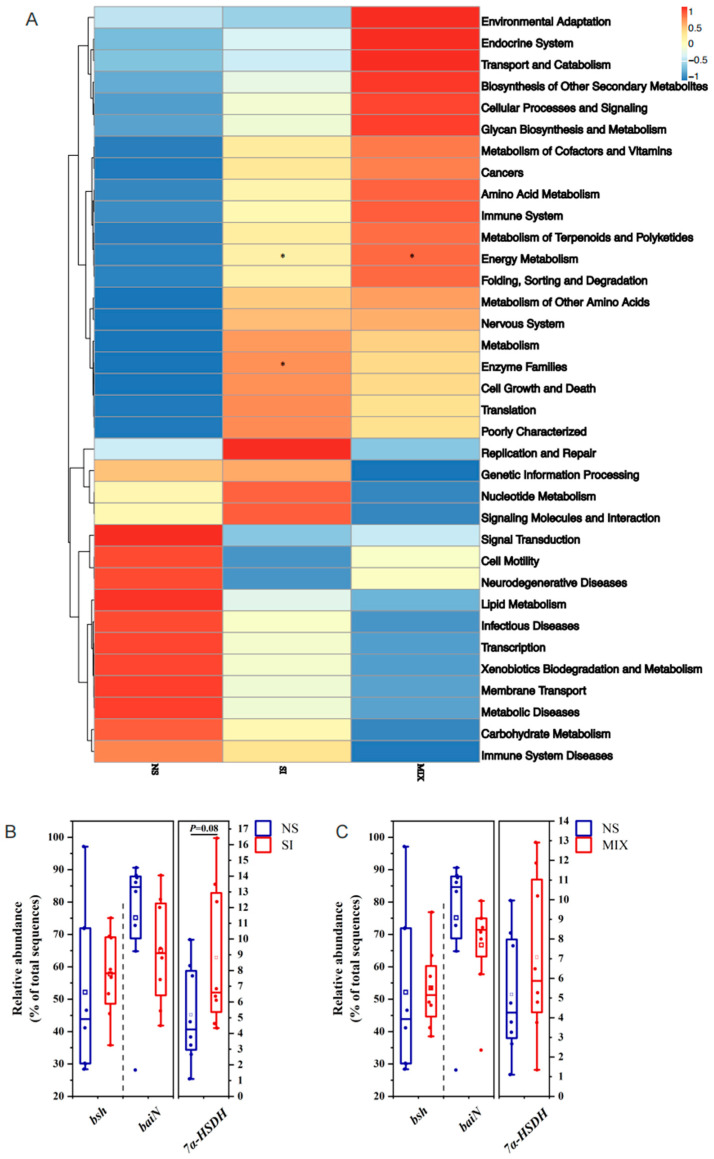
Effect of different treatment on the function of gut microbiota in piglets. (**A**) Heatmap of the gut microbiota function. (**B**,**C**) Functional difference analysis of colonic microbiota between the NS and SI group or the NS and MIX group. *, *p* < 0.05.

From Figure 4, the 3% SI or MIX diets increased the bacterial alpha diversity (Simpson’s index) in the colon (*p* < 0.05). *UCG-002* and *Holdemanella* were enriched in the SI group, while Bacteroidetes was enriched in the MIX group (Figure 4). Compared with the NS group, SI supplementation altered the KEGG pathway related to enzyme families and tended to elevate the relative abundance of the gut bacteria capable of expressing 7α-hydroxysteroid dehydrogenase (7α-HSDH) (*p* = 0.08) (Figure 5).

## 4. Discussion

Interventional studies in animals have shown that dietary fibers consisting of insoluble and soluble fiber, which are widely distributed carbohydrates in nature, can regulate the growth, immunity, and metabolism of animals [1,26,27]. In the swine industry, a disorder in lipid metabolism will lead to excessive lipid accumulation in pigs, which negatively impacts the lean meat rate and carcass quality [6]. Previous studies have demonstrated that dietary fiber has abilities to reduce lipid accumulation in growing-finishing pigs [7,8]. In this study, compared with NS, SI reduced the serum TC concentration and ADG, while MIX reduced the serum level of total bile acids in piglets. This finding is in line with previous research, which showed fructooligosaccharides can effectively lower the blood lipid levels in mice or growing-finishing pigs [28,29,30]. These results imply that dietary fiber can affect lipid metabolism in weaning pigs, but the precise effects and underlying mechanisms are not yet clear. Additionally, previous studies presented that insoluble fibers can disturb the reabsorption of bile acids in the intestine [31]. This might explain the observed impact of mixed fibers on the total bile acid levels.

Bile acids are derived from hepatic cholesterol, and the conversion of cholesterol to bile acids is facilitated by the enzymes CYP27A1 and CYP7A1 [13]. Some researchers have proposed that dietary intervention can affect the bile acid synthetic pathway and then lipid metabolism in the liver [32]. Thus, we analyzed the hepatic gene expression related to bile acid synthesis and lipid synthesis, and in this study, SI and MIX decreased the gene expression of CYP27A1, SREBP-1C, and ChREBP. SREBP-1c and ChREBP represent the critical regulators of lipid synthesis [33]. Li found that fructooligosaccharide down-regulated the levels of the SREBPs and ChREBP in mice fed a high sucrose diet [34]. Huang et al. noted that inulin reduced the gene expression of CHREBP, SREBP-1C, and FASN in mice fed a high-fat diet [35]. These findings align with our research, suggesting that dietary fiber possesses the ability to inhibit hepatic lipid synthesis. However, it remains unclear how dietary fiber affects hepatic lipid synthesis and blood lipid levels. As mentioned above, some secondary bile acids are associated with the regulation of hepatic lipid metabolism [20,36,37]. HDCA can downregulate the expression of *SREBP-1c*, *ACC*, and *FAS*, then alleviating NAFLD [20]. Zhong et al. also showed that dietary HDCA supplementation ameliorated NAFLD by activating fatty acid oxidation in mice [37]. Thus, a targeted metabolomics method was used to detect the composition and content of bile acids in piglets. The data showed that SI increased the level of THCA and α-MCA, and MIX increased the level of THCA and cholic acid. The level of GDCA was decreased in the SI and MIX group. The correlation analysis results showed that GDCA and TDCA were significantly positively correlated with TC. GDCA and TDCA, as secondary bile acids, are formed by gut microbiota and then enter the circulatory system [38]. A consistent study proposed that high dietary fiber intake of subjects was accompanied with the decrease in level of secondary bile acids (TDCA) in an Ovarian Cancer Screening Trial [39]. Jiao et al. (2018) also noted that the serum absolute concentrations of DCA and percent quantity of serum DCA were increased in patients with NAFLD [40]. In addition, Zheng et al. has suggested that the levels of HCA and its derivatives in those who are overweight/obese and overweight/obese with type 2 diabetes mellitus (T2DM) were significantly decreased, and HCA species were negatively correlated with blood glucose levels and diabetes [41,42]. In this study, there is a high concentration of α-MCA and THCA in pigs fed dietary fiber. THCA, a bile acid binding agent of taurocholic combined with HCA, belongs to the HCA or HDCA species [43]. HDCA and HCA can be converted from α-MCA via the enzymes encoded by gut microbiota [44]. Previous studies have demonstrated that HCA or HDCA species are closely related to hepatic lipid metabolism [20,41]. This implies that the regulatory effect of dietary fibers on lipid metabolism may be associated with secondary bile acids and the gut microbiota.

In addition to being involved in bile acid conversion, the enzymes encoded by gut microbes can also degrade nutrients, such as protein or dietary fiber [45,46]. To explore the potential relationship between dietary fibers and bile acids, this study conducted 16S rRNA gene sequencing to investigate the gut microbiota. The results demonstrated that SI or MIX increased the bacterial alpha diversity (Simpson’s index) in the colon. Xu et al. similarly found that fructooligosaccharides enhanced the alpha diversity of intestinal bacteria in mice fed a high-fat and high-cholesterol diet [47]. The bacterial genus *Holdemanella* has been identified as a potential contributor to health [48]. Previous research found a positive correlation between *Holdemanella* and the consumption of fermented dairy products, carbohydrates, and fiber [49]. Bacteroidetes, which are highly prevalent in the gut microbiota, are linked with the production of secondary bile acids [50]. Huang presented that supplementation of inulin can increase the relative abundance of Bacteroidetes [51]. Our results are similar to those obtained in previous studies; we found that *UCG-002* and *Holdemanella* were enriched in the SI group, and Bacteroidetes was enriched in the MIX group. The PICRUSt 2 function prediction was performed in order to analyze the microbial function. SI supplementation altered the KEGG pathway related to enzyme families, and there was a tendency to increase the relative abundance of gut bacteria capable of expressing 7α-HSDH in the SI group. As mentioned earlier, HCA species were converted from α-MCA through 7α-HSDH, 6β-hydroxysteroid dehydrogenase, and so on [52,53]. This suggests that fructooligosaccharides probably regulate the levels of bile acids through the gut microbiota and microbially produced enzymes.

## 5. Conclusions

Based on the results, we found that a dietary fiber mixture or fructooligosaccharides are able to increase the levels of HCA species and reduce the GDCA level in piglets. Dietary fiber supplementation, especially with fructooligosaccharides, can reduce the lipid synthesis in piglets, which may be related to the changes in the bile acids and gut microbiota.

## Figures and Tables

**Table 1:** The composition and nutrient levels of basal diet for weaning pigs. (%, as-fed basis).

Items	Content	Nutrient Level ^3^	
Corn starch	61.90	DE (MJ/kg)	16.07
Fish meal	5.00	Crude Protein	19.05
Soybean protein isolate (90%)	17.80	Calcium	0.75
Whey powder	8.00	Total phosphorus	0.52
Lactose	2.00	Lysine	1.29
Sucrose	3.00	Methionine	0.38
dl-Methionin (99%)	0.08	Tryptophan	0.26
l-Lysine-HCl (78%)	0.05	Threonine	0.76
l-Threonine	0.01		
l-Tryptophan	0.01		
Limestone	0.80		
Choline chloride (50%)	0.15		
NaCl	0.25		
Ca(H_2_PO_4_)_2_	0.70		
Vitamin premix ^1^	0.05		
Mineral premix ^2^	0.20		
Total	100.00		

^1^ Vitamin premix provided the following per kg of diets: vitamin A 9000 IU, vitamin D_3_ 3000 IU, vitamin E 20 IU, vitamin K_3_ 3.00 mg, vitamin B_1_ 1.50 mg, vitamin B_2_ 4.00 mg, vitamin B_6_ 3.00 mg, vitamin B_12_ 0.02 mg, biotin 0.10 mg, pantothenic acid 15 mg, nicotinic acid 30 mg, folic acid 0.75 mg. ^2^ Mineral premix provided the following per kg of diets: Fe (FeSO_4_) 100.00 mg, Cu (CuSO_4_) 25.00 mg, Zn (ZnSO_4_) 100.00 mg, Mn (MnSO_4_) 20.00 mg, Se (Na_2_SeO_3_) 0.30 mg, I (KI) 0.30 mg. ^3^ Nutrient levels were calculated levels.

**Table 2:** Sequences of the primers used for reverse transcriptase PCR analyses ^1^.

Genes	Primers	A_T_^c^ (°C)	Size, bp
*SREBP-1c*	F: AAGCGGACGGCTCACAATG R: GCAAGACGGCGGATTTATTCA	56.03	121
*FAS*	F: GCCGAGTACAGCGTCAACAACC R: TGGTCCTTCTTCATCAGCGGGAT	59.18	173
*ACACA*	F: CAACAATGGCATCGCAGCAGTG R: GGCTTTCAGGTCTTCGGGTGTG	58.44	121
*ChREBP*	F: ACAGACGCCTACACCTTCAAACTTC R: CCAGGACCCCACTGCTAAGGAC	58.19	83
*Cyp7a1*	F: GCATTTGGGCACAGAAGCATTGAC R: GGCAAGCAAATTCAAGGCGTCAC	58.67	105
*Cyp27a1*	F: AGAGTCATGGTACCGGCTGC R: AGAGCATTGGTGTAGAGCGCA	56.96	81
*FXR*	F: GACCACGAAGATCAGATTGCTTTGC R: ATGTCCAGCCGGAAGTTTCCTATTG	57.86	99
*β-actin*	F: TCTGGCACCACACCTTCT R: TGATCTGGGTCATCTTCTCAC	53.01	114

^1^ A_T_^c^, annealing temperature; *SREBP-1c*, sterol regulatory element-binding protein-1c; *FAS*, fatty acid synthase; *ACACA*, acetyl-CoA carboxylase; *ChREBP*, carbohydrate response element binding protein; *Cyp7a1*, cholesterol 7 alpha-hydroxylase; *Cyp27a1*, sterol 27-hydroxylase; *FXR*, farnesoid X receptor.

**Table 3:** Effect of different treatment on growth performance of weaning pigs ^1^.

Items	NS	SI	MIX	SEM	*p*-Value
1–21 d					
Initial weight, kg	10.53	10.52	10.53	0.23	1.00
Final weight, kg	21.00	17.92	19.32	0.54	0.06
ADFI, g/d	826	691	774	31.18	0.21
ADG, g/d	486 ^b^	357 ^a^	421 ^ab^	18.42	0.01
F/G	1.70 ^b^	1.94 ^a^	1.85 ^ab^	0.04	0.02

^1^ Eight piglets per treatment. Mean values followed by different letters within a row are significantly different (*p* < 0.05). ADFI, average daily feed intake; ADG, average daily gain; F/G, feed-to-gain ratio.

**Table 4:** Effect of different treatment on serum biochemical parameters of weaning pigs ^1^.

Items	NS	SI	MIX	SEM	*p*-Value
TG (mmol/L)	0.38	0.32	0.36	0.02	0.47
TC (mmol/L)	1.32	1.05	1.14	0.05	0.08
TBA (μmol/L)	17.99 ^b^	13.17 ^ab^	11.47 ^a^	1.18	0.04
LDL-C (mmol/L)	4.67	6.09	4.54	0.59	0.50
HDL-C (mmol/L)	3.29	3.18	3.52	0.13	0.57

^1^ Eight piglets per treatment. Mean values followed by different letters within a row are significantly different (*p* < 0.05). TG, total triglyceride; TC, total cholesterol; TBA, total bile acids; LDL-C, low density lipoprotein cholesterol; HDL-C, high density lipoprotein cholesterol.

**Table 5:** Effect of different treatment on bile acid profiles in bile of weaning pigs (μg/mL) ^1^.

Items	NS	SI	MIX	SEM	*p*-Value
THCA	223.11 ^a^	354.10 ^b^	411.84 ^b^	28.57	0.01
GDCA	703.93 ^b^	457.13 ^a^	442.85 ^a^	43.55	0.02
GCA	439.85 ^a^	582.92 ^ab^	712.13 ^b^	43.65	0.03
UDCA	1.18 ^ab^	2.79 ^b^	0.45 ^a^	0.39	0.04
α-MCA	12.35	42.56	13.02	6.16	0.07
GUDCA	996.62	1147.11	707.29	86.01	0.10
TMCA	13.08	20.92	25.38	2.48	0.12
GHDCA	31,557.63	33,730.90	26,025.04	1674.02	0.15
12-KLCA	1.74	1.25	0.44	0.28	0.16
7-KDCA	27.85	28.93	20.61	1.96	0.17
DCA	1.96	0.46	0.20	0.42	0.19
HDCA	109.77	120.17	25.92	24.63	0.24
TCA	171.22	163.29	231.20	17.86	0.25
TDCA	24.97	16.28	20.55	5.01	0.28
GCDCA	6661.24	7018.93	6216.24	212.85	0.32
LCA	0.33	0.16	0.13	0.06	0.32
ω-MCA	12.35	18.57	11.77	2.03	0.33
CDCA	12.79	11.19	4.36	2.52	0.37
GLCA	212.31	132.88	158.87	24.02	0.41
TLCA	7.88	5.31	5.76	0.86	0.44
TUDCA	45.83	70.77	65.24	8.20	0.45
CA	41.53	47.62	23.69	9.32	0.57
THDCA	1656.30	1495.12	1447.70	107.11	0.73

^1^ Eight piglets per treatment. Mean values followed by different letters within a row are significantly different (*p* < 0.05). THCA, taurohyocholic acid; GDCA, glycodeoxycholic acid; GCA, glycocholic acid; UDCA, ursodeoxycholic acid; α-MCA, α-muricholic acid; GUDCA, glycoursodeoxycholic acid; TMCA, tauromuricholic acid; GHDCA, glycohyodeoxycholic acid; 12-KLCA, 12-ketolithocholic acid; 7-KDCA, 7-ketodeoxycholic acid; DCA, deoxycholic acid; HDCA, hyodeoxycholic acid; TCA, taurocholic acid; TDCA, taurodeoxycholic acid; GCDCA, glycochenodeoxycholic acid; LCA, lithocholic acid; ω-MCA, ω-muricholic acid; CDCA, chenodeoxycholic acid; GLCA, glyco lithocholic acid; TLCA, taurolithocholic acid; TUDCA, tauroursodeoxycholic acid; CA, cholic acid; THDCA, taurohyodeoxycholic acid.

## Data Availability

The data underlying this article will be shared on reasonable request to the corresponding author.
